# Choroidal Structural Changes Assessed with Swept-Source Optical Coherence Tomography after Cataract Surgery in Eyes with Diabetic Retinopathy

**DOI:** 10.1155/2020/5839837

**Published:** 2020-10-29

**Authors:** Huiping Yao, Sha Gao, Xiaoqing Liu, Yufeng Zhou, Yu Cheng, Xi Shen

**Affiliations:** ^1^Department of Ophthalmology, Ruijin Hospital, Shanghai Jiao Tong University School of Medicine, Shanghai 200025, China; ^2^Department of Ophthalmology, Ruijin Hospital North, Shanghai Jiao Tong University School of Medicine, Shanghai 201801, China

## Abstract

**Objective:**

To determine the influence of phacoemulsification on choroidal vasculature in patients with diabetic retinopathy (DR) undergoing cataract surgery using swept-source optical coherence tomography (SS-OCT).

**Methods:**

The study was conducted in 23 eyes of 23 cataract patients with mild/moderate nonproliferative diabetic retinopathy (NPDR) without diabetic macular edema (DME) and 23 age-matched controls. Choroidal thickness (CT) and choroidal vascularity index (CVI) were measured at baseline and 1 week, 1 month, and 3 months after surgery.

**Results:**

The baseline CVI in the DR group was significantly lower than that in the control group (*P*=0.001). CVI in DR patients after surgery significantly increased compared with preoperative values (all *P* < 0.001 for 1 week, 1 month, and 3 months after surgery). Postoperative increase of CVI and CT in the DR group was more than in the control group, and the difference was significant 1 month and 3 months after surgery (all *P* < 0.05).

**Conclusion:**

Patients with mild/moderate NPDR have reduced CVI compared with nondiabetic patients at baseline; diabetic cataract surgery tended to induce more increase in CVI and CT as compared with nondiabetic patients. This trial is registered with NCT04499768.

## 1. Introduction

Diabetes is considered to be a risk factor of cataract incidence and development; diabetic patients usually develop cataracts earlier [1]. Cataract extraction using phacoemulsification usually results in remarkable visual acuity outcomes and is one of the most common and safe ophthalmic surgical procedures. However, diabetic cataract surgery sometimes could be complicated by macular edema, and progression of diabetic retinopathy [2, 3], and these complications also lead to poor visual acuity recovery, so the right timing of cataract surgery of diabetic patients should be considered.

The choroid is one region with high blood circulation ratio, predominantly composed of blood vessels and surrounding stromal tissue. Choroid is the major blood supply to the retina, supplying nutrients and oxygen to the retina. Thus, choroid with normal structure and function is important for maintaining normal retinal physiology [4, 5]. Diabetic choroidopathy (DC) was firstly reported by Hidayat and Fine, and it was reported that there existed arteriosclerotic choroidal arteries, luminal narrowing, basement membranes thickening, and choriocapillaris dropout in DC [6]. It was pointed out that DC may play a role in the pathology of DR [6, 7].

With the advent of optical coherence tomography (OCT) technology, choroidal thickness (CT) was widely measured and it was noted that most patients with senile cataract were expected to maintain increased choroid thickness at least for several months after cataract surgery [8–10]. Recently, CVI is used to distinguish lumens of choroidal vasculature from surrounding stroma and has been proposed as a marker for vascular health of choroid [11–15]. Choroidal vasculature might be involved in the diabetic eyes and change with duration of diabetes, and a reduced CVI has been recently noted in DR [13, 16]. But the influence of the cataract surgery on the choroidal vascular structures in DR patients is not clear.

The new generation swept-source optical coherence tomography (SS-OCT) system penetrates deeper into the tissue and provides high-resolution images, so it is thought to have advantages for the assessment of the choroid [17, 18]. In this study, we aimed to determine the influence of phacoemulsification on choroidal vascular structures in patients with DR undergoing cataract surgery through the follow-up values of CT and CVI using this SS-OCT system.

## 2. Methods

### 2.1. Study Subjects

This study included 23 eyes of 23 cataract patients with mild/moderate NPDR without DME, and 23 age-matched nondiabetic patients were also enrolled in this study. All individuals underwent comprehensive ophthalmologic examination, including a dilated fundal examination, measurements of IOP, best-corrected visual acuity (BCVA), axial length (AL), and SS-OCT. IOP was measured using noncontact tonometry (TX-20, Canon, Japan) at all visits; BCVA was measured using a standard logarithmic visual acuity chart to calculate the logarithm of reciprocal decimal visual acuity (logMAR VA) at baseline and 3 months after surgery; AL was measured using ocular biometry (LenStar LS-900, Haag-Streit AG, Switzerland) at baseline; and SS-OCT was measured at all visits. The stage of NPDR was graded based on the international clinical disease severity scale for DR [19]. All cataract patients undergoing uncomplicated phacoemulsification surgery were recruited consecutively (from June 2018 to June 2019) from the Ophthalmology Department of Ruijin Hospital North and signed the consent forms. The study was adhered to the provisions of the Declaration of Helsinki and was approved by the Ethical Review Committee of Ruijin Hospital North. If both eyes of one patient were operated, only the eye which was operated on first was enrolled.

The inclusion criteria were as follows: aged >40 years, intraocular pressure (IOP) < 21 mmHg in both eyes, and spherical refractive error <6 diopters spherical equivalent. The exclusion criteria were as follows: previous retinal surgery, glaucoma, uveitis, age-related macular degeneration, arterial or vein occlusions, macular hole, or other ocular diseases that could interfere the CT and/or CVI measurement, severe systemic diseases, such as leukemia, rheumatic disease, malignant tumors, uncontrolled hypertension, obstructive sleep apnea, etc., and decreased media transparency precluding appropriate OCT imaging.

### 2.2. Cataract Surgery

The phacoemulsification cataract surgeries were performed by the same skilled surgeon using the Infinity Vision System (Alcon Laboratories, Inc.). None of the patients had operative complications. All patients had hydrophilic acrylic intraocular lens (Softec HD, Lenstec, Inc. USA) implanted into their capsular bags and had one month of topical steroids. The cumulative dissipated energy (CDE) was documented. Postoperative treatment consisted of tobramycin dexamethasone eyedrops administered 4 times a day for 1 week and then prednisolone acetate 1.0% and levofloxacin 0.5% eyedrops administered 3 times a day for 3 weeks.

### 2.3. SS-OCT Data Acquisition

All study subjects were imaged using the SS-OCT (Triton DRI-OCT, Topcon, Inc, Tokyo, Japan) preoperatively and at 1 week (W1), 1 month (M1), and 3 months (M3) postoperatively, and the OCT scans were tracked at all follow-up visits. All images were acquired in the morning considering the diurnal variation of choroid vasculature. Images with a signal strength index of more than 40 and no residual motion artifacts were saved for further analysis. CT was defined as the distance between the outer boundary of retinal pigment epithelium/Bruch's membrane and the chorioscleral interface, and the CT values were obtained with the built-in software of the SS-OCT device (Topcon FastMap, version 10.13.003.06). The foveal CT was defined as the CT value in the center circle in the standard ETDRS grid; the parafoveal CT was defined as the arithmetic average value of CT in subregions in the annulus with an outer diameter of 3.0 mm and an inner diameter of 1.0 mm in the standard ETDRS grid; the perifoveal CT was defined as the arithmetic average value of CT in subregions in the annulus with an outer diameter of 6.0 mm and an inner diameter of 3.0 mm in the standard ETDRS grid.

### 2.4. Procedures of Image Binarization

**Figure 1:**
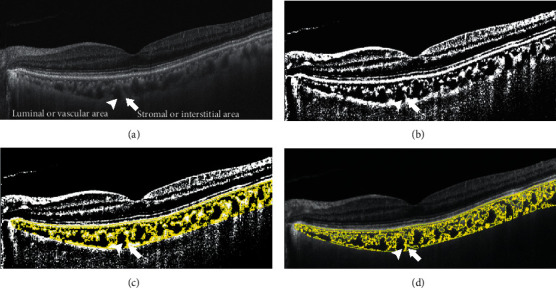
The dark pixels (arrow head) represented the luminal or vascular area and the light pixels (arrow) were defined as stromal or interstitial area. (a) SS-OCT scan image. (b) Image was converted with the auto local threshold tool. (c) The dark pixels were selected using the threshold tool. (d) An overlay image of ROI of the binarized segment of the choroid on SS-OCT scan.

CVI was acquired through the method described as follows. Images were analyzed by one public domain software, Image J (version 1.52a, provided in the public domain by the National Institutes of Health, Bethesda, MD, USA; http://imagej.nih.gov/ij/), using the protocol as previously described with modifications [11, 12, 20]. Briefly, 9 mm scan images passing through the fovea horizontally were chosen. The region of interest (ROI) was manually selected using the polygon tool and added to the ROI manager. The images were adjusted by the Niblack auto local threshold. The binarized image was converted to the RGB image, and the luminal area was determined using the threshold tool. The dark pixels represented the luminal or vascular area, and the light pixels were defined as stromal or interstitial area (Figure 1). CVI was defined as the proportion of vascular area to total circumscribed area.

### 2.5. Inter-Rater and Intra-Rater Agreement

Two independent graders masked from patient information initially segmented 46 images from all the subjects. The same images were segmented by one of the above two graders after an interval of one week to determine intra-rater reliability. The inter-rater reliability and intra-rater reliability for image binarization were indicated by the absolute agreement model of the intraclass correlation coefficient (ICC), performed using IBM SPSS statistics version 21.0. The ICC value of 0.81–1.00 indicates good agreement.

### 2.6. Statistical Analysis

Statistical analyses were performed using GraphPad Prism 7.0 (GraphPad Software, CA). Normally distributed data were expressed as mean ± standard deviation (SD). The preoperative and postoperative measurements were compared by the paired *t*-test. The difference between two groups was compared by the unpaired *t*-test. Pearson correlation analyses were performed to determine the relationships between CVI/CT and related factors. *P* < 0.05 was considered to be statistically significant.

## 3. Results

A total of 46 eyes of 46 subjects were recruited in the study; the DR group included 23 mild/moderate NPDR patients (12 males and 11 females) and the control group included 23 nondiabetic patients (13 males and 10 females).  Table 1 shows patients' demographic and clinical characteristics, and the DR group included 14 moderate NPDR patients and 9 mild NPDR patients; 3 patients had coronary heart disease and 7 patients had well-controlled hypertension in the DR group. There was no significant difference in age, gender, BCVA, IOP, AL, or CDE between the DR group and the control group.

After the cataract surgery, the BCVA was significantly improved and IOP significantly decreased. The baseline IOP in DR and control groups was 16.1 ± 0.6 mmHg and 15.6 ± 0.6 mmHg, respectively. After surgery, the IOP of the control group significantly decreased (*P*=0.019, <0.001, and <0.001 for 1 week, 1 month, and 3 months after surgery compared with the preoperative values). After surgery, the IOP of the DR group also significantly decreased (*P*=0.002, <0.001, and <0.001 for 1 week, 1 month, and 3 months after surgery compared with the preoperative values).

### 3.1. Inter-Rater and Intra-Rater Agreement for CVI

The inter-rater agreement for CVI was 0.932 (95% confidence interval (CI): 0.877–0.962) for average measure and 0.872 (95% CI: 0.781–0.927) for single measure; the intra-rater agreement for CVI was 0.951 (95% CI: 0.911–0.973) for average measure and 0.906 (95% CI: 0.837–0.947) for single measure. These ICC values above indicated a significantly high agreement for the image segment.

### 3.2. CVI

**Figure 2:**
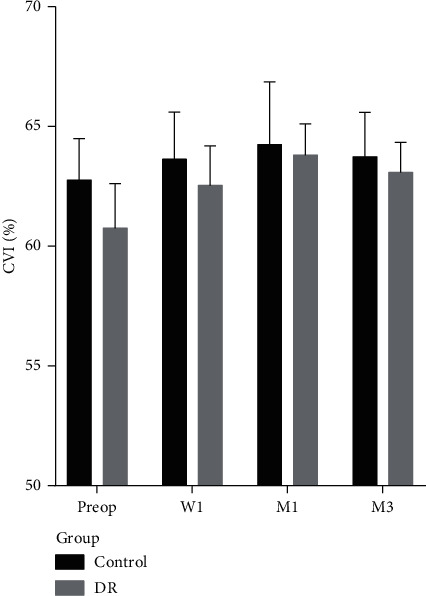
The choroidal vascularity index for patients with mild/moderate NPDR (DR) and nondiabetic patients (control) at the four visits (mean ± SEM). W1: 1 wk postop; M1: 1 mo postop; M3: 3 mo postop.

The baseline CVI in the DR group was significantly lower than that in the control group; there was a significant difference between the two groups (mean difference = 2.00% and 95% CI: 0.94%–3.07%). After surgery, the CVI of the control group was significantly increased at 1 month and 3 months postoperatively. The CVI of the DR group significantly increased at 1 week, 1 month, and 3 months postoperatively (Table 2 and Figure 2). CVI at any visit did not correlate with age, AL, CDE, or IOP in the DR group and control group (Supplementary Table 1).

**Figure 3:**
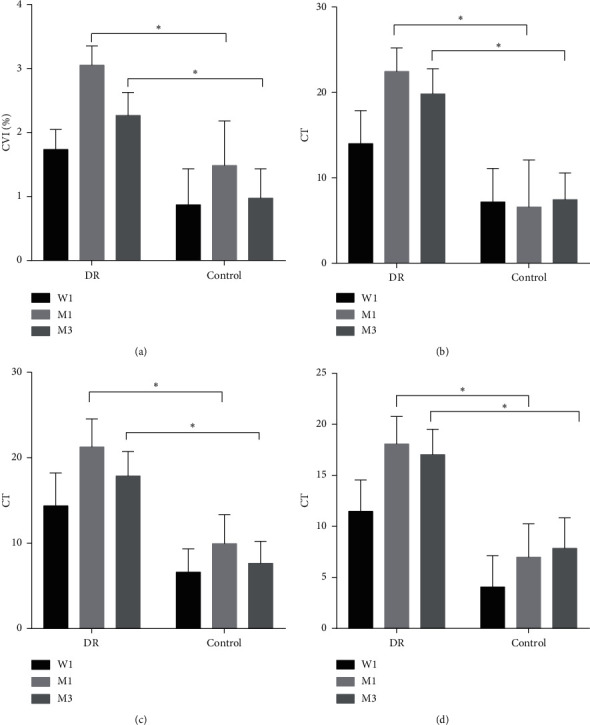
(a) Changes of CVI, (b) changes of foveal CT, (c) changes of parafoveal CT, and (d) changes of perifoveal CT for patients with mild/moderate NPDR (DR) and nondiabetic patients (control) at 1 week, 1 month, and 3 months postoperatively (mean ± SEM). W1: 1 wk postop; M1: 1 mo postop; M3: 3 mo postop. ^*∗*^*P* < 0.05.

After surgery, increase of CVI in DR group was more than that in the control group, and the difference was significant at 1 month and 3 months after surgery (mean difference = 1.57%; 95% CI: 0.04–3.09% for 1 month after surgery; mean difference = 1.29%; 95% CI: 0.12%–2.46% for 3 months after surgery) (Table 3 and Figure 3).

### 3.3. CT

The baseline CT of the fovea, parafovea, and perifovea of the DR group was not significantly thinner than that of the control group (*P*=0.423, 0.254, and 0.133 for fovea, parafovea, and perifovea, respectively).

**Figure 4:**
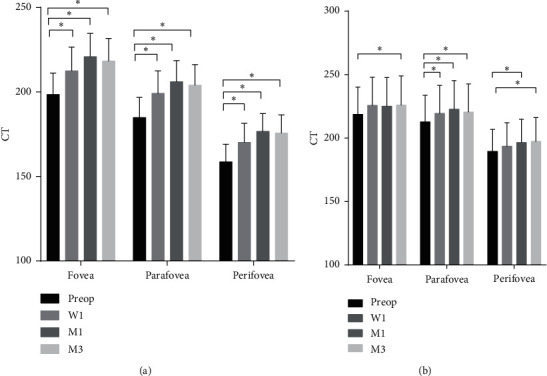
The choroidal thickness of fovea, parafovea, and perifovea for patients with mild/moderate NPDR (a) and nondiabetic patients (b) at four visits (mean ± SEM) W1: 1 wk postop; M1: 1 mo postop; M3: 3 mo postop. ^*∗*^*P* < 0.05.

A significant increase in CT was found after surgery in the DR group. The CT of the DR group significantly increased at 1 week, 1 month, and 3 months postoperatively in all regions (Figure 4 and Table 4).

In the control group, CT was found to increase in some parts of the macula. The CT of fovea at 3 months postoperatively, the CT of parafovea at 1 week, 1 month and 3 months postoperatively, and the CT of perifovea at 1 month and 3 months postoperatively were found significantly thicker than the baseline CT (Figure 4 and Table 4). CT did not correlate with AL, CDE, or IOP at any region in the control group and DR group (Supplementary Tables 2 and 3).

After surgery, increase of CT in the DR group was more than that in the control group, 1 month, and 3 months after surgery in all regions of macula (Table 3 and Figure 3). The difference between the two groups was significant for 1 month and 3 months after surgery at fovea (mean difference = 15.86; 95% CI: 3.46–28.26 for 1 month after surgery; mean difference = 12.39; 95% CI: 3.77–21.01 for 3 months after surgery); the difference was also significant for 1 month and 3 months after surgery at parafovea (mean difference = 11.33; 95% CI: 1.81–20.84 for 1 month after surgery; mean difference = 10.22; 95% CI: 2.42–18.02 for 3 months after surgery) and was significant for 1 month and 3 months after surgery at perifovea (mean difference = 11.1; 95% CI: 2.57–19.62 for 1 month after surgery; mean difference = 9.17; 95% CI: 1.34–17.01 for 3 months after surgery).

## 4. Discussion

Cataract extraction has been identified as a risk factor for DR progression [21, 22]. However, less DR progression was reported in patients undergoing phacoemulsification as compared with intracapsular cataract extraction and extracapsular cataract extraction [23], and Squirrell et al. even concluded that uncomplicated phacoemulsification cataract surgery does not cause acceleration of diabetic retinopathy postoperatively [24]. There seems to exist some controversies.

In the present study, we focused on quantitatively evaluating changes of the choroidal structures through SS-OCT-based CT and CVI measurements in patients with DR and controls undergoing phacoemulsification, aiming to make clear the possible influence of cataract surgery on changes of choroidal structure in patients with DR, which might play an important role in the pathogenesis of DR.

CVI has been found to be a relatively stable index to monitor the progression of choroidal diseases [13, 25, 26] and provide us more information on the proportion of vascularity in the choroid [11]. The significantly low CVI might reflect the narrowing and/or closure of choroidal vessels. In this study, baseline CVI was found to be significantly lower in patients with mild/moderate NPDR as compared with the controls, which seemed to be a hint of the existence of diabetic vasculopathy in DR patients, despite the result that no significant difference had been found between DR patients and controls in the baseline CT in this study. These results were similar to the previous studies [13, 16]. Tan et al. have reported a significantly lower CVI in diabetic patients with DR and without DR as compared with controls, but they found no significant differences in the CT between patients with DM and controls [13]. CVI seems to provide us more information on diabetic vasculopathy in DR and be more sensitive in monitoring the changes of choroidal vascularity in DR.

The influence of surgery on the choroidal structure is uncertain in the diabetics in previous studies. A number of publications have reported that phacoemulsification may cause a significant increase in choroid thickness [27–29]. However, some studies found no CT variation after cataract surgery in diabetics [30, 31].

In our study, CVI of the patients with mild/moderate NPDR was found to increase significantly at all visits after surgery; in contrast, the CVI of the control group had no significant increase 1 week after surgery, but it was found to increase 1 month and 3 months after surgery; therefore, the increase of CVI seemed to occur earlier in the patients with mild/moderate NPDR than in the controls. In addition, we also found that the variation in CVI and CT before and after surgery in patients with mild/moderate NPDR was more than that in the controls 1 month and 3 months after surgery. Obviously, for the first time, our present study showed that diabetic cataract surgery tended to induce more increase in the CVI and CT as compared with the controls. All the results above implied that cataract surgery seemed to have more influence on the choroidal vascularity in mild/moderate DR patients than in nondiabetic patients.

It has been experimentally verified that cataract surgery induces the expression of some proinflammatory cytokine in the choroid; it was detected that IL-1*β* and CCL2 gene expression and protein expression were upregulated in the choroid [32]. In addition, the upregulation of vascular endothelial growth factor (VEGF) was observed in the diabetic choroid, and VEGF may contribute to increased vascular permeability and angiogenesis during retinopathy [33]. The high levels of proinflammatory cytokines and VEGF in the preoperative choroid of patients with DR may be one of the underlying mechanisms of the upregulating expression postoperatively, which is likely to induce more increase in CT and CVI in mild/moderate NPDR patients than in nondiabetic patients.

In addition, the increased ocular perfusion pressure caused by reduced IOP may induce increased CT in the early period after phacoemulsification [28]. In our study, the IOP of patients with mild/moderate NPDR and controls both significantly decreased after surgery and no significant difference existed between the two groups. The reduced IOP might not be one of the underlying causes of the difference in the variation of CT and CVI following cataract surgery between the patients with mild/moderate NPDR and nondiabetic patients.

One limitation of our study is the relatively small sample size, but the individuals enrolled in had homogeneous ethnicity and similar gender distribution, which reduced some confounding effects. Another limitation is that the follow-up time is short, so the long period influence on choroid could not be observed in this study. A long-time follow-up should be needed in future studies to make clear how long the increase in CVI and CT following cataract surgery would last. Therefore, a large population sample with a longer follow-up should be recommended in future studies.

## 5. Conclusion

For the first time, the present study showed that cataract surgery tended to induce more increase of CVI and CT in patients with mild/moderate NPDR as compared with nondiabetic patients. Therefore, the cataract surgery seemed to have more influence on the choroid vasculature in DR patients than in nondiabetic patients, which might imply the underlying impact of cataract surgery on the progression of the choroidopathy in DR patients. Meanwhile, baseline CVI was found to be significantly lower in DR patients as compared with the nondiabetic patients. Therefore, CVI could be reckoned as a biomarker for vascular health of choroid in DR patients and might have the potential to be a predictor of progression of DC following cataract surgery. Furthermore, whether changes of CVI and/or CT could be the predictors of progression of DR following cataract surgery at subclinical stages should be focused on in future studies.

## Figures and Tables

**Table 1:** Demographic and clinical characteristics of the eyes included for analysis.

		Control (*n* = 23)	DR (*n* = 23)	*P* value
Age, years (mean ± SD)		67.0 ± 9.5	66.2 ± 7.6	0.758
Gender, *n* (%)	Male	13 (56.5)	12 (52.2)	0.767
	Female	10 (43.5)	11 (47.8)	
BCVA, log MAR (mean ± Sd)	B	0.56 ± 0.17	0.54 ± 0.14	0.58
	M3	0.04 ± 0.09	0.03 ± 0.1	0.665
IOP, mmHg (mean ± SD)	B	15.6 ± 2.8	16.1 ± 2.7	0.574
	W1	13.8 ± 3.8	14.2 ± 2.7	0.672
	M1	13.7 ± 2.8	13.8 ± 2.5	0.974
	M3	13.5 ± 2.5	13.7 ± 2.6	0.763
AL, mm (mean ± SD)		23.3 ± 1.0	23.2 ± 1.0	0.794
CDE (mean ± SD)		6.4 ± 4.6	5.7 ± 4.4	0.616
FBS, mmol/L (mean ± SD)		NA	7.48 ± 1.00	NA
HbA1c, % (mean ± SD)		NA	7.36 ± 0.78	NA
Cr, *μ*mol/L (mean ± SD)		NA	80.39 ± 15.99	NA

B: baseline; W1: 1 wk postop; M1: 1 mo postop; M3: 3 mo postop; CDE: cumulative dissipated energy; AL: axial length; SD: standard deviation; FBS: fasting blood sugar; HbA1c: glycated hemoglobin A1c; Cr: creatinine.

**Table 2:** CVI of the patients with mild/moderate NPDR (DR) and nondiabetic patients (control) at four visits (mean ± SD).

	Baseline	Postop					
		1 wk postop		1 mo postop		3 mo postop	
	CVI	CVI	*P* value	CVI	*P* value	CVI	*P* value
Control (%)	62.77 ± 1.73	63.64 ± 1.97	0.136	64.25 ± 2.61	0.044^*∗*^	63.74 ± 1.86	0.044^*∗*^
DR (%)	60.76 ± 1.85	62.5 ± 1.8	<0.001^*∗*^	63.82 ± 1.3	<0.001^*∗*^	63.03 ± 1.54	<0.001^*∗*^

^*∗*^ *P* < 0.05. SD: standard deviation.

**Table 3:** Changes of CVI and CT after cataract surgery (mean ± SD).

		W1			M1			M3		
		Control	DR	*P* value	Control	DR	*P* value	Control	DR	*P* value
CVI (%)		0.871 ± 2.697	1.736 ± 1.495	0.204	1.486 ± 3.331	3.051 ± 1.436	0.044^*∗*^	0.974 ± 2.19	2.267 ± 1.72	0.035^*∗*^
CT (*μ*m)	Fovea	7.174 ± 18.85	14.00 ± 18.48	0.222	6.587 ± 26.36	22.45 ± 13.27	0.013^*∗*^	7.435 ± 14.95	19.83 ± 14.05	0.006^*∗*^
	Parafovea	6.609 ± 13.11	14.37 ± 18.52	0.108	9.935 ± 16.25	21.26 ± 15.76	0.021^*∗*^	7.641 ± 12.36	17.86 ± 13.84	0.011^*∗*^
	Perifovea	4.065 ± 14.66	11.46 ± 14.82	0.096	6.978 ± 15.66	18.08 ± 12.89	0.012^*∗*^	7.848 ± 14.33	17.02 ± 11.92	0.023^*∗*^

W1: 1 wk postop; M1: 1 mo postop; M3: 3 mo postop. *∗P* < 0.05 . SD: standard deviation.

**Table 4:** The choroid thickness of the patients with mild/moderate NPDR (DR) and nondiabetic patients (control) at four visits (mean ± SD).

		Baseline	Postop					
			1 wk postop		1 mo postop		3 mo postop	
		CT	CT	*P* value	CT	*P* value	CT	*P* value
Control (*μ*m)	Fovea	217.6 ± 105.2	223.3 ± 108.1	0.106	223.8 ± 109.2	0.269	224.7 ± 112	0.031^*∗*^
	Parafovea	213.8 ± 99.9	221.9 ± 104.4	0.025^*∗*^	224.2 ± 106.6	0.006^*∗*^	221.8 ± 105.7	0.006^*∗*^
	Perifovea	189.6 ± 82.9	193.6 ± 88.5	0.2	196.5 ± 88.2	0.044^*∗*^	197.4 ± 90	0.015^*∗*^
DR (*μ*m)	Fovea	198.4 ± 61.2	212.4 ± 68.2	0.002^*∗*^	220.9 ± 66.4	<0.001^*∗*^	218.3 ± 64.3	<0.001^*∗*^
	Parafovea	184.9 ± 57.3	199.2 ± 63.7	0.001^*∗*^	206.1 ± 60.1	<0.001^*∗*^	204.1 ± 58.7	<0.001^*∗*^
	Perifovea	158.7 ± 50.1	170.1 ± 54.6	0.001^*∗*^	176.7 ± 50.1	<0.001^*∗*^	175.7 ± 51.6	<0.001^*∗*^

^*∗*^ *P* < 0.05. SD: standard deviation.

## Data Availability

All the relevant data of this study are available from the corresponding author upon request.

## Supplementary Materials

Supplementary Materials Supplementary Table 1: correlations of CVI with AL, CDE, and IOP for patients with mild/moderate NPDR (DR) and nondiabetic patients (control) at four visits. Supplementary Table 2: correlations of CT with AL, CDE, and IOP for patients with mild/moderate NPDR at four visits in fovea, parafovea, and perifovea. Supplementary Table 3: correlations of CT with AL, CDE, and IOP for nondiabetic patients at four visits in fovea, parafovea, and perifovea.
