# Supplementary material for: Choroidal Structural Changes Assessed with Swept-Source Optical Coherence Tomography after Cataract Surgery in Eyes with Diabetic Retinopathy
Source: J Ophthalmol. 2020 Oct 29;2020:5839837. doi: 10.1155/2020/5839837 (PMC7648249; doi:10.1155/2020/5839837)
Supplement: Supplementary Materials — Supplementary Table 1: correlations of CVI with AL, CDE, and IOP for patients with mild/moderate NPDR (DR) and nondiabetic patients (control) at four visits. Supplementary Table 2: correlations of CT with AL, CDE, and IOP for patients with mild/moderate NPDR at four visits in fovea, parafovea, and perifovea. Supplementary Table 3: correlations of CT with AL, CDE, and IOP for nondiabetic patients at four visits in fovea, parafovea, and perifovea. [file 5839837.f1.docx]

Supplementary Table 1. Correlations of CVI with AL, CDE, and IOP for patients with mild/moderate NPDR (DR) and non-diabetic patients (control) at four visits.

|  | | age | | AL | | CDE | | IOP | |
| --- | --- | --- | --- | --- | --- | --- | --- | --- | --- |
|  |  | r | *p*-value | r | *p*-value | r | *p*-value | r | *p*-value |
| DR | B | 0.077 | 0.726 | -0.276 | 0.202 | 0.257 | 0.237 | -0.146 | 0.506 |
|  | W1 | 0.196 | 0.370 | -0.104 | 0.638 | -0.043 | 0.845 | -0.082 | 0.709 |
|  | M1 | -0.205 | 0.347 | -0.099 | 0.652 | -0.015 | 0.948 | -0.025 | 0.910 |
|  | M3 | -0.223 | 0337 | -0.318 | 0.140 | 0.158 | 0.471 | 0.127 | 0.449 |
| control | B | -0.174 | 0.427 | -0.346 | 0.106 | -0.105 | 0.632 | -0.146 | 0.506 |
|  | W1 | 0.032 | 0.887 | 0.048 | 0.829 | -0.128 | 0.561 | 0.046 | 0.836 |
|  | M1 | 0.158 | 0.472 | -0.334 | 0.119 | -0.046 | 0.834 | -0.151 | 0.491 |
|  | M3 | 0.195 | 0.372 | 0.021 | 0.923 | 0.026 | 0.908 | -0.167 | 0.447 |

B: baseline; W1:1 Wk Postop; M1: 1 Mo Postop; M3: 3 Mo Postop; CDE: cumulative dissipated energy; AL: axial length; IOP: intraocular pressure; CDE: cumulative dissipated energy

Supplementary Table 2. Correlations of CT with AL, CDE, and IOP for patients with mild/moderate NPDR at four visits in fovea, parafovea and perifovea.

|  | | age | | AL | | CDE | | IOP | |
| --- | --- | --- | --- | --- | --- | --- | --- | --- | --- |
|  |  | r | *p*-value | r | *p*-value | r | *p*-value | r | *p*-value |
| fovea | B | -0.109 | 0.646 | 0.084 | 0.725 | 0.042 | 0.88 | 0.127 | 0.562 |
|  | W1 | -0.047 | 0.844 | 0.070 | 0.77 | 0.004 | 0.985 | 0.075 | 0.733 |
|  | M1 | -0.061 | 0.900 | 0.049 | 0.837 | 0.015 | 0.947 | 0.193 | 0.378 |
|  | M3 | -0.109 | 0.898 | 0.029 | 0.903 | 0.079 | 0.719 | -0.048 | 0.829 |
| parafovea | B | -0.197 | 0.405 | 0.175 | 0.461 | 0.024 | 0.913 | 0.195 | 0.373 |
|  | W1 | -0.098 | 0.681 | 0.130 | 0.584 | -0.042 | 0.849 | 0.124 | 0.510 |
|  | M1 | -0.120 | 0.615 | 0.132 | 0.579 | -0.014 | 0.947 | 0.218 | 0.319 |
|  | M3 | -0.145 | 0.542 | 0.104 | 0.662 | 0.030 | 0.893 | 0.009 | 0.968 |
| perifovea | B | -0.206 | 0.383 | 0.263 | 0.262 | -0.021 | 0.923 | 0.188 | 0.391 |
|  | W1 | -0.099 | 0.679 | 0.217 | 0.3576 | -0.091 | 0.678 | 0.135 | 0.538 |
|  | M1 | -0.155 | 0.513 | 0.233 | 0.323 | -0.049 | 0.825 | 0.219 | 0.316 |
|  | M3 | -0.206 | 0.536 | 0.226 | 0.339 | -0.031 | 0.889 | -0.001 | 0.996 |

B: baseline; W1:1 Wk Postop; M1: 1 Mo Postop; M3: 3 Mo Postop; CDE: cumulative dissipated energy; AL: axial length; IOP: intraocular pressure; CDE: cumulative dissipated energy

Supplementary Table 3. Correlations of CT with AL, CDE, and IOP for non-diabetic patients at four visits in fovea, parafovea and perifovea.

|  | | age | | AL | | CDE | | IOP | |
| --- | --- | --- | --- | --- | --- | --- | --- | --- | --- |
|  |  | r | *p*-value | r | *p*-value | r | *p*-value | r | *p*-value |
| fovea | B | -0.255 | 0.266 | -0.206 | 0.345 | 0.202 | 0.357 | 0.039 | 0.860 |
|  | W1 | -0.287 | 0.185 | -0.228 | 0.295 | 0.204 | 0.350 | 0.069 | 0.754 |
|  | M1 | -0.321 | 0.135 | -0.223 | 0.306 | 0.051 | 0.816 | -0.116 | 0.598 |
|  | M3 | -0.257 | 0.236 | -0.216 | 0.333 | 0.179 | 0.414 | -0.090 | 0.684 |
| parafovea | B | -0.252 | 0.247 | -0.166 | 0.449 | 0.223 | 0.307 | 0.044 | 0.843 |
|  | W1 | -0.242 | 0.266 | -0.179 | 0.415 | 0.254 | 0.242 | 0.051 | 0.816 |
|  | M1 | -0.271 | 0.212 | -0.183 | 0.402 | 0.220 | 0.312 | -0.225 | 0.302 |
|  | M3 | -0.230 | 0.291 | -0.177 | 0.419 | 0.237 | 0.275 | -0.105 | 0.633 |
| perifovea | B | -0.252 | 0.246 | -0.121 | 0.582 | 0.238 | 0.275 | 0.049 | 0.823 |
|  | W1 | -0.239 | 0.271 | -0.165 | 0.452 | 0.246 | 0.257 | 0.034 | 0.876 |
|  | M1 | -0.247 | 0.256 | -0.140 | 0.524 | 0.247 | 0.256 | -0.265 | 0.222 |
|  | M3 | -0.231 | 0.288 | -0.138 | 0.529 | 0.234 | 0.282 | -0.145 | 0.509 |

B: baseline; W1:1 Wk Postop; M1: 1 Mo Postop; M3: 3 Mo Postop; CDE: cumulative dissipated energy; AL: axial length; IOP: intraocular pressure; CDE: cumulative dissipated energy
